# Supplementary figures and images for: Genomic Resources for the First Federally Endangered Lichen: The Florida Perforate Cladonia (Cladonia perforata)
Source: J Fungi (Basel). 2023 Jun 24;9(7):698. doi: 10.3390/jof9070698 (PMC10381751; doi:10.3390/jof9070698)

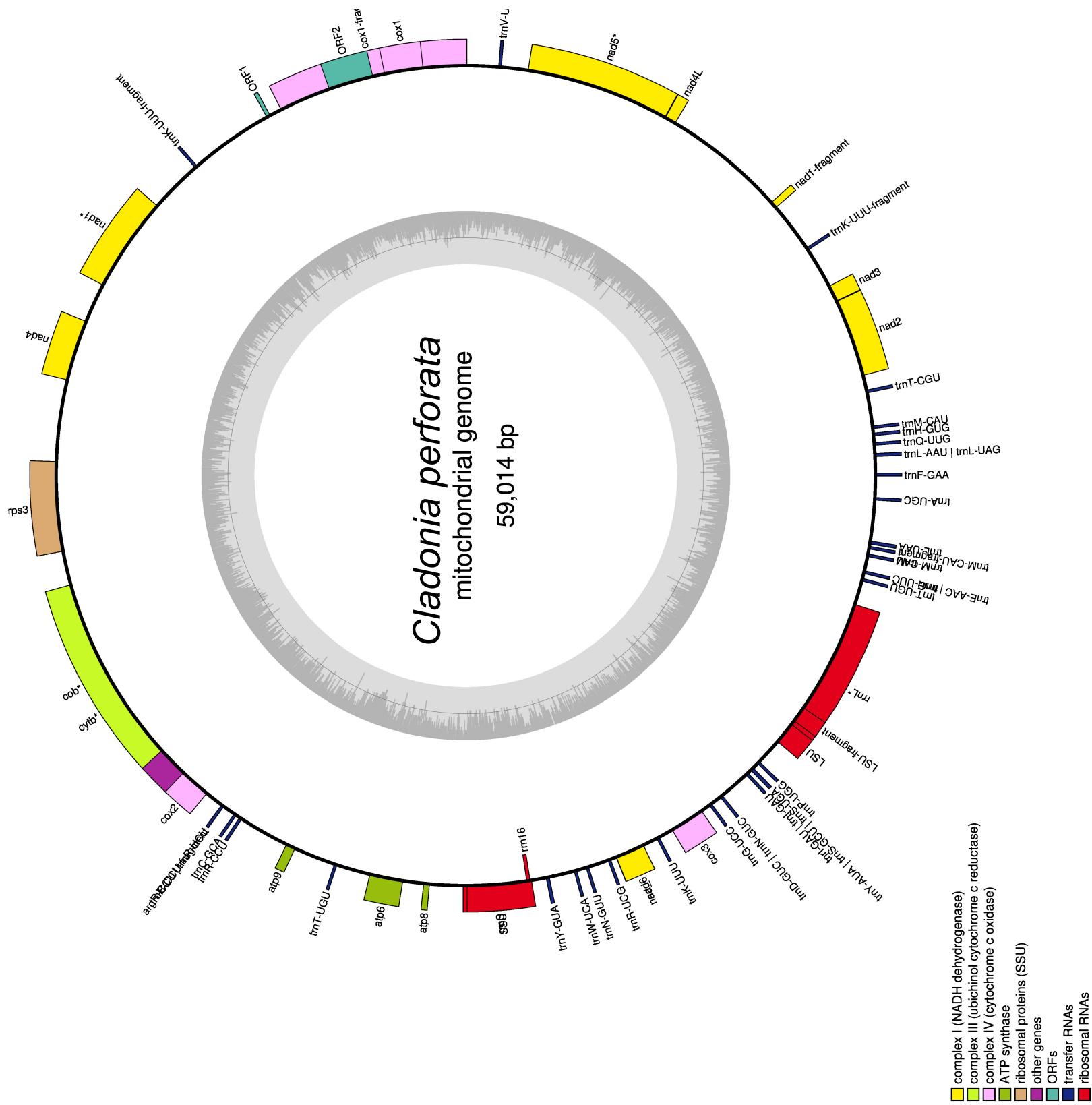

Supplement: Supplementary file 1 [file jof-09-00698-s001.zip › S4_cladonia_perforata_mt_genome_orig_OGDRAW.pdf]

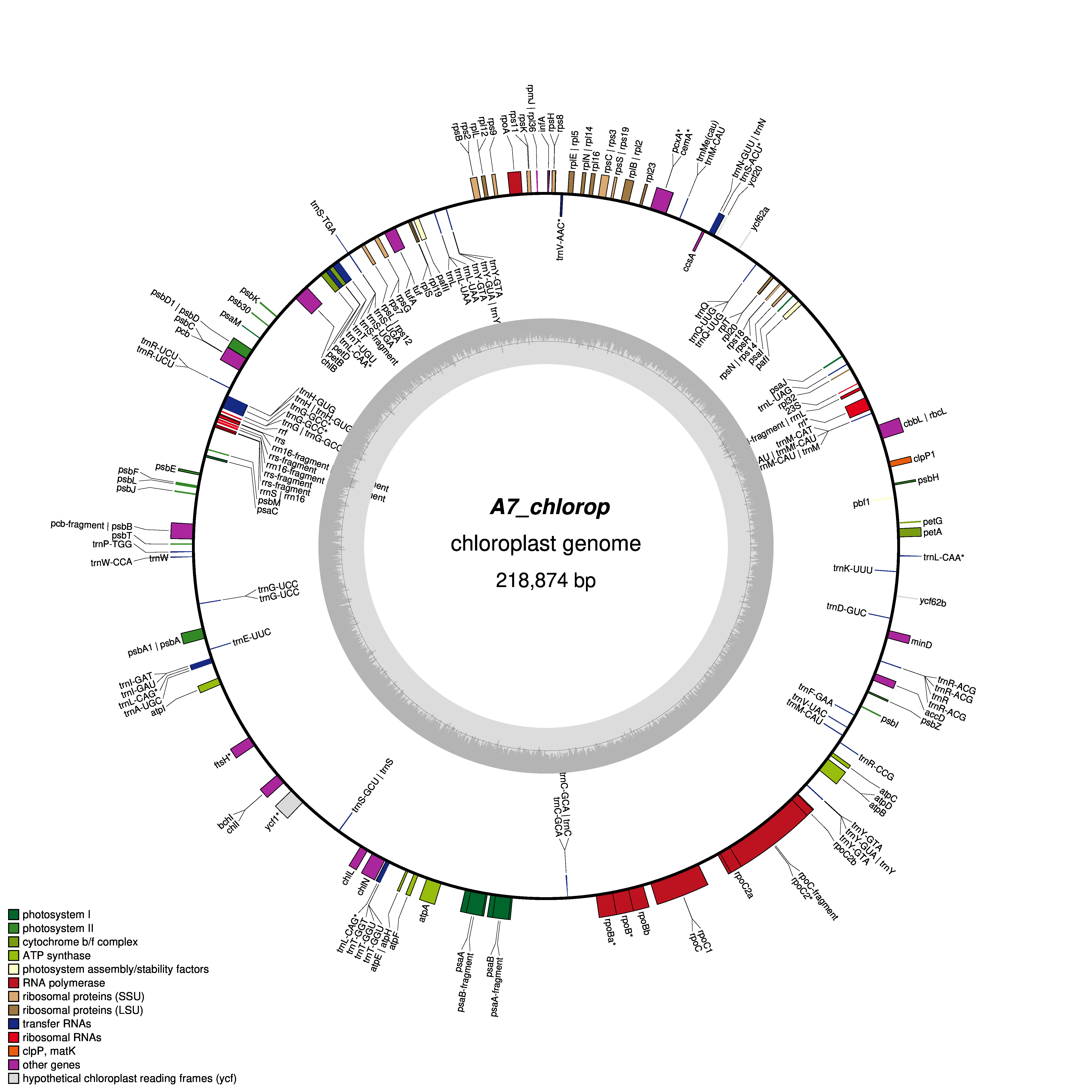

Supplement: Supplementary file 1 [file jof-09-00698-s001.zip › S6_GeSeqJob-20230405-121348_A7_chloroplast_genome_OGDRAW.jpg]
